# Irritable Bowel Syndrome between Molecular Approach and Clinical Expertise—Searching for Gap Fillers in the Oxidative Stress Way of Thinking

**DOI:** 10.3390/medicina56010038

**Published:** 2020-01-19

**Authors:** Ioana-Miruna Balmus, Ovidiu Ilie-Dumitru, Alin Ciobica, Roxana-Oana Cojocariu, Carol Stanciu, Anca Trifan, Mirela Cimpeanu, Cristian Cimpeanu, Lucian Gorgan

**Affiliations:** 1 Department of Interdisciplinary Research in Science, Alexandru Ioan Cuza University of Iasi, Carol I Avenue, No. 11, 700506 Iasi, Romania; balmus.ioanamiruna@yahoo.com; 2 Department of Biology, Faculty of Biology, Alexandru Ioan Cuza University of Iasi, Carol I Avenue, 20A, 700506 Iasi, Romaniacristiansorin.cimpeanu@gmail.com (C.C.);; 3 Department of Research, Faculty of Biology, Alexandru Ioan Cuza University of Iasi, Carol I Avenue, 20A, 700506 Iasi, Romania; 4 Center of Biomedical Research, Romanian Academy, 8th Carol I Avenue, 700506 Iasi, Romania; stanciucarol@yahoo.com; 5 Department of Gastroenterology, Faculty of Medicine, “Gr. T. Popa” University of Medicine and Pharmacy, 16th University Street, 700115 Iasi, Romania

**Keywords:** irritable bowel syndrome, oxidative stress, inflammatory status, predisposition genes, animal models

## Abstract

Irritable bowel syndrome (IBS) remains to date an intriguing functional gastrointestinal disorder. Recent studies described a multitude of exogenous factors that work together in IBS, gradually impairing intestinal lining cellular metabolism, including oxidative status balance, with or without a genetic background. Although the current biomarkers support the differentiation between IBS subtypes and other functional gastrointestinal disorder, they are mostly non-specific, referring to clinical, biochemical, and inflammatory imbalances. Since IBS could be also the result of deficient signaling pathways involving both gastrointestinal secretion and neuro-vegetative stimulation, IBS makes no exception from the oxidative hypothesis in the pathological mechanisms. Regarding the oxidative stress implication in IBS, the previous research efforts showed controversial results, with some animal models and patient studies reporting clear oxidative imbalance both on systemic and local levels, but still with no concrete evidence to point to a direct correlation between oxidative stress and IBS. Additionally, it seems that a major role could be also attributed to gut microbiota and their ability to shape our bodies and behaviors. Moreover, the genetic features study in IBS patients showed that several genetic similarities point to a possible correlation of IBS with affective spectrum disorders. Thus, we focus here the discussion on the assumption that IBS could in fact be more likely a stress-related disorder rather than a gastrointestinal one.

## 1. Introduction

To date, scientific knowledge on irritable bowel syndrome (IBS) include extensive information regarding diagnostic criteria, clinical symptomatology, classification, and risk factors. However, a detailed description of molecular diagnostic biomarkers or complete etiology and origin of the disease are particularities yet to be treated. Despite that, the prevalence of IBS is rather high, between 11% and 21% of the global population [1], statistics that differ, for example, in diabetic patients [2].

According to the newest classifications in gastroenterology diseases [3], IBS is currently categorized as functional gastrointestinal impairment comprising several key intestinal symptoms. The most common clinical manifestations of IBS are the frequent intestinal habits changes, being defined mainly through stool consistency variations (constipation, diarrhea, or alteration on account of lack of an exact clinical cause such as intestinal inflammation or infection) [4]. Further symptomatology includes abdominal pain and discomfort which improves with defecation, also in the absence of a pathological triggering factor. However, only around 30% of the people experiencing IBS symptomatology are consulting a physician, many of whom are women [4].

In this way, the origin of IBS is not fully understood, but several possible causes and risk factors include the age-related stress occurring between the first 20 to 40 years of life (intense activity and higher incidence in daily and occupational stress), female sex (however not clear why twice as many women than men are diagnosed with IBS, due to menstrual cycle hormonal changes or a more responsible attitude towards pathological body changes), the familial history of IBS (despite that no clear correlation between IBS and genetic factors was established), or unbalanced or gastrointestinal tract-aggressive diet (high incidence of irritant spices or foods such as hot spices, neurostimulants, certain vegetables, and dairy products) [1,4,5,6,7].

Considering the incidence of the mentioned symptomatology and risk factors, and not knowing the exact pattern of interaction between genetic and environmental components, IBS endues several clinical variants and was categorized according to the main feature. In this way, considering stool consistency, IBS was categorized in constipation-predominant IBS (C-IBS), diarrhea-predominant IBS (D-IBS), and mixed IBS (M-IBS). Except for these three main clinical variants, research efforts defined another three IBS clinical variants such as pain-predominant IBS (P-IBS), post-infectious IBS (PI-IBS), and post-diverticulitis IBS (PD-IBS). Any other gastrointestinal manifestations that meet the IBS criteria could be diagnosed as unclassified IBS (U-IBS), according to ROME III diagnosis criteria [8].

However, according to ROME IV criteria, functional gastrointestinal disorders received a new definition. In this way, functional gastrointestinal disorders are now disorders of gut–brain interaction, a vast group of gastrointestinal disorders, which include common symptoms such as motility disturbance, visceral hypersensitivity (VH), altered mucosal and immune function, altered gut microbiota, and altered central nervous system processing [9]. Moreover, some of the ROME III criteria were actualized and a more specific diagnosis standard was established. Several changes regarding the symptoms frequency and definition were imperious, since the interactions between some of the IBS symptoms were not entirely correlated (e.g., pain occurrence in correlation to stool changes or defecation) [10].

Since ROME IV has given a new valence to the IBS diagnosis by introducing the brain-gut axis alterations into the definition [9], in this review we mainly aimed to open the discussion regarding the possible molecular diagnosis of IBS, despite the non-invading tissular features of IBS.

## 2. Making Room for Molecular Approach 

Almost considering it an unwritten rule, molecular approaches must start from clear, understandable, and reproducible molecular parameters which are changed in a specific manner in a studied disease. Present knowledge in IBS reported no specific IBS molecular parameter, such as the already common decreased blood haemoglobin for anemia or increased serum aminotransferases for hepatitis, since IBS has no specific or clearly deducible histopathological effect. Most of the current biomarkers regarding IBS diagnosis do not refer to direct diagnosis, but rather to differentiating IBS from other diseases. In this way, besides the eight-item immunological biomarker panel described by Mujagic et al. [11], which proposed and validated interleukin 1-beta, interleukin 6, interleukin 12p70, tumour necrosis factor-α, chromgranin A, human beta-defensin 2 (HBD2), calprotectin, and caproate in a multitest IBS panel, many studies described differentiating biomarkers. Still, the reports are controversial since some of those biomarkers were also previously described as non-specific, and the panel was validated against a healthy controls’ cohort. For instance, calprotectin which is a degradation-resistant calcium and zinc-binding protein involved in intestinal inflammatory response was mentioned in this panel, but also identified in IBD patients [12]. Similarly, serum HBD2 was also reported as possible biomarker in several inflammatory skin conditions such as psoriasis [13,14], and several gastrointestinal inflammatory diseases such as ulcerative colitis, Crohn disease, necrotizing enterocolitis, and bacterial intestinal infections [15].

Recent studies regarding non-invasive IBS biomarkers described several biochemical parameters through respiratory chemical analysis of IBS patients. In this way, some differences in gas composition of the exhaled air were reported, as compared to healthy controls, as a result of altered intestinal activity and gut microbiota composition [16]. The study mentioned that aziridines were not previously detected in other biological samples than feces, but it seems that antibiotic treatment-induced intestinal bacterial overgrowth in IBS patients thrived in aziridine producers such as *Actinobacteria*. However, the authors highlighted the fact that none of the IBS patients included in their study underwent antibiotic treatment, therefore, they suggest that aziridines origin in exhaled air is purely speculative. Despite that, Zhuang et al. [17] released an altered gut microbiota species list which also includes members of the *Actinobacteria* phylum.

Thus, finding some reliable diagnosis markers in IBS is rather a matter of distinguishing IBS from other known and well-studied diseases, which are similar to IBS in symptomatology. Since it was described that IBS does not stand out through prominent intestinal nor systemic changes, we could speculate that the specific IBS diagnosis molecular parameter might be correlated to other IBS components rather than the gastrointestinal/biochemical/inflammatory ones. Supporting this assumption is the new definition for functional gastrointestinal disorders according to which it seems that IBS might be a matter of molecular impairment rather than functional unbalance. For instance, several recent studies suggested that IBS is correlated to some molecular changes which occur before or concomitant to IBS symptomatology [18,19,20].

In this way, the molecular basis of IBS is closely tied to visceral hyperalgesia, intestinal hyper-permeability, gut microbiome composition, psychosocial distress, food intolerance, colonic bacterial fermentation, genetics, and gut inflammation [21]. However, as recent studies showed, the biopsychosocial model approach in IBS diagnosis and management could be the most effective due to the increased molecular multifactorial character of IBS [21,22].

Moreover, as individual differences in IBS symptomatology are rather common, the true challenge is to formulate a clear molecular picture. In this way, current research efforts are trying to manage the vast molecular features of IBS in the absence of a clear cause-effect relationship, using animal models and functional and molecular explorations in IBS patients. 

## 3. Molecularly Different, But Clinically the Same

For better understanding of IBS molecular components, however, it is important to consider the differences and similarities that coexist in the diverse IBS subtypes. Since it seems that the molecular pathways underlying the diarrhea and constipation—the two major features of IBS symptomatology and categorization—a multifactorial understanding of the molecular changes occurring in IBS pathogenesis could help find the disease origin.

One of the most consistent lines of evidence that the intestinal symptomatology origin mechanism is quite different could be represented by the comparison of the IBS subtypes predominant with constipation and diarrhea considering the implication of bile acids in IBS pathology. Even though bile acids play an important role in both C-IBS and D-IBS, the stool consistency changes are only partly explained, as suggested by Camilleri et al. [19]. In this way, taking into consideration the correlation between the bile acid variation and stool consistency, the gut microbiota could also interfere in bile acid mechanisms of action due to its capacity to deconjugate primary bile acids and therefore alter their signaling.

Additionally, regarding to gut microbiota activity, several studies suggested that the mechanism of diarrhea in IBS could be associated with the potential of microbial species in carbohydrate fermentation which is further correlated to increased serotonin release (by short-chain fatty acids signaling), as we will describe in the next section of our present report [23]. Even though constipation and diarrhea undergo different molecular mechanisms, many molecular biomarkers regarding the mucosa permeability showed significant increases in both IBS-C and IBS-D, as compared to healthy controls [24]. Thus, distribution and expression of the proteins consisted of similar mucosa tight junctions’ changes in IBS-C and IBS-D which suggested a third-party mucosa permeability regulation system [24].

Another important molecular feature of IBS is the altered visceral sensation. Many mediators and receptors including neurotransmitter receptors, cannabinoid receptors, opioid receptors, gamma-aminobutyric acid receptors, glutamate receptors, glucocorticoid receptors, inflammatory receptors, and ion channel receptors are implicated in visceral sensation processing, and several psychosocial factors [23]. Several studies also showed that IBS visceral hypersensitivity is well correlated to emotional instability [25], and descending pain modulatory system impairment [26].

Thus, disregarding some differences, IBS development could be more a matter of similarity than difference. In this way, the possibility that IBS-C and IBS-D could be two different syndromes is not sustained by scientific evidence based on the assumption that different molecular pathways equal different diseases. Moreover, since IBS occurs developing both diarrhea and constipation—in some cases in the same person—defined as mixed-IBS or alternating-IBS, it seems that the key mechanism of action could not be linked with intestinal distress in bowel movement or mucosa permeability. However, the most important piece of evidence highlighted in this constipation-diarrhoea comparison in IBS is the fact that in both symptoms neuroenteric abnormalities and microbiota changes were reported.

## 4. It Is a Bug Situation

Gut microbiota is a crucial component for all living organisms mainly because of the deficient enzymatic function in glyosidic linkage [27]. Moreover, their extensive ability to produce energy needed to maintain vital functions through enzymatic pathways involved in the digestion of carbohydrates and proteins both expands and helps the regulation of the host metabolism [27]. Thus, anaerobic or aerobic symbiotic bacteria, such as the colonic communities of individuals from *Faecalibacterium*, *Bacteroides*, *Enterobacteria*, and *Bifidobacterium* genera species stimulates indigestible oligosaccharides resulting the synthesis of short-chain fatty acids (SCFAs): acetate, butyrate, and propionate [28]. Furthermore, bacterial catabolism includes secondary products, such as hydrogen sulphide, methane, and other metabolites which could play a promoter or inhibitor role in bacterial and host genetic expression [29,30]. 

In this way, whereas butyrate is usually involved in blocking the accumulation of toxic byproducts, for example D-lactate [31], in downregulating inflammatory reactions [32] and even prevent colon carcinogenesis [33], acetate and propionate cross the enteric blood barrier to participate in gluconeogenesis and lipogenesis as substrates [34]. 

Another important mechanism in which the microbiota is involved is the production of micronutrients, such as vitamin K (*Enterobacter agglomerans*, *Serratia marcescens*, and *Enterococcus faecium* [35]) and menaquinone (actively involved in decreasing vascular calcification by regulating its deposition and improving osteocalcin circulation [35]). Intestinal microbiota also plays an important role in vitamin B complex synthesis, including pantothenic acid (B5) and cyanocobalamin (B12), the latter being a well-known modulator of acetylcholine and cortisol production in adrenal gland. Both these vitamins were reported in deficient levels in gastrointestinal, neuropsychological, and hematological disorders [36,37]. Regarding the serotonin enteric metabolism actively implicated in gastrointestinal motility regulation, it was recently shown that gut microbiota participates, alongside the enterochromaffin cells, to its production [38]. In this way, microorganisms such as *Bacteroides* spp., altered Schaedler flora, and spore-forming *Clostridium* species were described to be serotonin producers during intestinal colonization [38].

Regarding the bacterial species distribution in IBS, minor differences were observed in fecal bacteria distribution of *Lactobacillus*, *Coccidioides*, *Clostridium*, and *Faecalibacterium prausnitzi* species [39]. However, IBS-D patients were reported with significantly increased presence of *Escherichia coli* colonies, as well as significantly decreased presence of *Clostridium leptum* and *Bifidobacterium* sp. [39]. Additionally, the significant implication of unconjugated bile salt hydrolases in enteric nervous system deregulations were previously described by Dey et al. [40], in a gnotobiotic mouse model to transient specific dietary supply. Moreover, it seems that the proliferation of certain SCFAs-producing species leads to water and electrolyte circuit changes inducing abdominal bloating and distension [41]. Furthermore, it was shown that bile acids could also modulate 7α-hydroxy-4-cholesten-3-one synthesis and release which could alterate bowel habits by accelerating colonic transit and determining dysentery and visceral hypersensitivity [42].

A recent meta-analysis which summarized 13 studies up to 2016 revealed that significant features defined each group between IBS and healthy controls for *Lactobacillus*, *Bifidobacterium*, and *Faecalibacterium prausnitzii*. On the other hand, no important differences were noted among *Bacteroides-Prevotella* group, *Enterococcus*, *Escherichia coli*, or *Clostridium coccoides* within these patients. In IBS-D patients, *Lactobacillus* and *Bifidobacterium* strains’ concentration is lower compared with IBS-C, with the higher prevalence in Chinese subjects [43,44]. A study conducted by using cohorts from Iberian and Scandinavian Peninsula with different ages and diagnosed according to Rome II and III criteria concluded that dysbiosis was detected in 73% of IBS patients, as compared to healthy controls in which dysbiosis was 16% [45]. The main contributors were represented by *Firmicutes* phylum (*Bacillus* and *Ruminococcus gnavus*), *Proteobacteria* (*Shigella* or *Escherichia*), and *Actinobacteria*. 

It has also been shown that IBS symptoms’ severity is negatively associated with microbial richness and have distinctive signatures [46]. In another two studies, it was found that *Pseudomonas aeruginosa* is also a participant, indicating a link between this Gram-negative bacillus and IBS [47,48]. Some variable clues due to the heterogenous nature of the disease, particularities of study design, different protocols used for sample collection and size, and data analysis are represented by a decreased alpha diversity; however, this might not be nonspecific, since it has also been reported in other gastrointestinal deficiencies such as obesity, Crohn’s disease, ulcerative colitis, and necrotizing enterocolitis [49,50,51,52].

Furthermore, additional surveys indicate increased levels of families belonging to *Bacteroidetes* phylum [27,53], *Giardia duodenalis* constituting one of the commonly related post-infections parasites in IBS, microbiota’s biofilm indicating a reduction from 100–210 to 10–105 µM [54]. Lyra et al. [29] designed a study with the aim of distinguishing IBS subtypes by dividing volunteers into three categories: IBS-D, IBS-C, and IBS-M. They discovered that above 80% of total strains identified were detected in all samples analyzed. Through real-time PCR increased amounts of *Veillonella* spp. [30] were also shown, in parallel with a decrease ratio of *Bifidobacterium catenulatum* [31] and *Methanobrevibacter smithii* [32]. By amplifying variable regions V1-V3 and V6 of the 16S rRNA gene and subsequently sequenced, Caroll et al. [33] showed that IBS-D’s microbial composition is altered. Culture analysis of intestinal samples (fecal and colonic samples) revealed a significant reduction in the concentration of aerobic bacteria in fecal samples [34]. 

Thus, we summarized the microbiota diversity in different IBS-subtypes in this section, as seen in Table 1.

In IBS-D and IBS-M patients, the number of butyrate-producing bacteria is diminished activating nociceptive sensory pathways while IBS patients that do not undergo any therapy had a lower abundance of *Methanobacteria* [55]. Moreover, IBS is responsible for a petulant production of SCFAs which contribute to visceral pain responsivity and motility, specific for IBS [34,56]. Commensal microbes within the colon metabolize prebiotics (food supplements administered for the growth and/or activity of beneficial strains) in order to produce acetate, butyrate, and propionate, most of lactic bacteria having the ability to utilize oligofructose [37]. These fatty acids bind to nicotinic acid receptors (G-protein coupled receptors) GPR43, GPR41, and GPR109A with a known capacity to reduce inflammatory reactions in several tissues such as, brain, vascular, and adipose [36]. Supplementary evidence that consolidates this bidirectional pathway is represented by a significant decrease of serum oxytocin and cortisol in patients with major depression and IBS [38,57].

Regarding the similarities of IBS with affective disorders in terms of microbiota, it was shown that depressed patients exhibiting altered gut microbiota composition also showed signs of abnormalities in hypothalamic–pituitary–adrenal axis function, low-grade intestinal inflammation, and brain–gut–microbiota axis neurotransmitter metabolism impairment [58]. Similarly, the depressed patients’ fecal microbiota transplantation in germ free mice led to depression-like behaviors in a probiotic reversable manner, recent studies showed [59,60]. Additionally, anxiety-like behaviors could be modulated by intestinal microflora inhibition, in a mice germ-free model [61]. In this way, these findings could explain why IBS diversity and stability of gut microbiota affected patients also exhibit anxious and depressive-like behaviors. Moreover, a recent study showed that the mycobiome impairment occurring in IBS could be the major cause of visceral hypersensitivity [62]. Thus, while the molecular mechanisms implicated in the cause-effect relationship between gut microbiota and mood and behavior phenotypes are not fully understood, we have reasons to believe that a major role in both IBS and mood and behavior regulation could be attributed to gut microbiota. 

## 5. When Things Get Inflamed

Under normal circumstances, the intestinal epithelium forms the active intestinal lumen–blood barrier in which the immune responses’ main purpose is to maintain the integrity of the normal symbiosis. Commensal bacteria prevent the adherence of pathogens, train the immune system, and protect intestinal epithelial cells (IECs) [63], while Paneth cells prevent the penetration of intestinal epithelium [64]. However, when tight junctions are breached by influx of inflammatory mediators, harmful microbes inside the gut induce intense immune reactions affecting structure and composition [65]. Considering that IECs are protected on the plasma surface by glycocalyx, immunoglobulin A, belt desmosomes, chloride secretion, and other glycoproteins [63,66], intestinal epithelium consists in more than 40 different types of proteins implicated in the homeostasis of a wide range of T cells, especially Th1 and Th17 [67]. Thus, the “leaky gut” triggers low-grade inflammatory responses in IBS in which are involved from T lymphocytes and mast cells to pro-inflammatory cytokines [68,69].

Since one out of 10 individuals with IBS think that their illness began with a previous exposure to pathogens, environmental predisposition gained a relative meaning, primarily depending on the infection type. Thus, the post-infectious IBS type was extensively studied even though IBS was previously believed to be a strictly functional disturbance [70]. A series of risk factors have been described by Thabane et al. [71], among which are the common ones of the 21st century, depression and anxiety.

According to ROME IV criteria, post-infectious IBS is defined as the exhibition of IBS symptomatology following treated gastrointestinal infections, closely resembling IBS-D in 5%–32% in post-acute gastroenteritis patients [72]. Usually, in post-infection stages, inhibitory potential of SCFAs against opportunistic new intruders is profoundly reduced, existing the presumption that acute gastroenteritis can induce to a small extent, sensitization of the small intestine if there are psychosocial factors that perturb the mast cells [73]. Species such as Salmonella, *Campylobacter jejuni*, or *Shigella* are among the first microorganisms identified in patients with specific symptomatology [42,74,75], recent data indicating 3% to 33% of all PI-IBS.

In PI-IBS, are characteristic high levels of Toll Like Receptor 4 (TLR-4) [76], enterochromaffin cells, lymphocyte, and interleukin-1β mRNA [77]. A relationship between several PI-IBS microbial markers and host’s amino acids has been recently documented [78]. Hydrogen sulphide is the result of fermentation between digestive microbiota and protein metabolism, and is one of the harmful products [79], with the capacity to impair intestinal epithelium by acting as a signaling molecule [80], which subsequently forms tetrathionate, promoting a later development of *Salmonella* [81].

It has been reported that both enterochromaffin cells and mast cells play a role in PI-IBS. Rectal biopsies revealed that infections with *Campylobacter jejuni* increased T lymphocytes—C3, CD4 and CD8 [82]—while Barbara et al. [83] observed in mucosal biopsies elevated levels of mast cell mediators. In this subgroup, lymphocytes are more reactive after bacterial stimulation. Interleukin-1β is one of them, whose activity is deregulated after an attack of bacillary dysentery [84]. Additionally, various cytokines (interleukin-2, -6, -10), transforming growth factor beta (TGF-β) [41], and an altered expression of tool-like receptors (TLRs) 2, 4, 7, and 8 [85,86,87] are specific for PI-IBS. 

Considering that IBS is a multifactorial disease with a characteristic clinical panel and pathological mechanisms still insufficiently understood, in order to distinguished IBS from other Functional Gastrointestinal Disorders (FGIDs) based on the notable differences highlighted to date, some of them will be discussed below [67].

One of the most important issues to consider is inflammation mismatch between IBS and IBD due to the fact that IBS lies somewhere in the spectrum of functionality while IBD is more an organic disorder, argument sustained by a characteristic mucosal inflammation and symptomatology that do not certify endoscopic conclusions [68], identification of reliable biomarkers being a priority and a major step forward for the management of this disease. It would be unrealistic to expect that a single candidate biomarker could be the answer to all questions, which is why intestinal tissue was the perfect starting point to search novel biomarkers, the same logic also being viable for IBS by using urine, stool, and blood samples [88]. Calcium-signaling heterodimer, calprotectin, constituted the cornerstone for non-invasively testing for IBS. However, since calprotectin is yet another non-specific and differential diagnosis biomarker, it helps clinicians, in the first step, to distinguish between non-inflammatory and inflammatory disease of the colon, as Caviglia et al. [89] suggested. Additionally, included in this category is erythrocyte sedimentation rate (ESR), levels of cortisol, and those of chromogranin.

C-reactive protein (CRP) is one of the several proteins synthetized by the liver whose activity is intensified as a response to factors released by macrophages, Hod et al. [90] using it as an indicator for IBS values of high-sensitivity CRP (hs-CRP). With a score of <100 µg/g cut off value, Jelsness-Jørgensen et al. [82] deduced that values under 40 µg/g are not indicators, while above 100 µg/g, marks a significant inflammation which points to IBD. ELISA tests were used with the aim to distinguish IBD from IBS, stool calprotectin having a pool sensitivity of 93% and 94% specificity at FC with a cut-off level of 50 µg/g for IBS, while the cut-off between 83% and 100% was associated with an ongoing inflammation [69]. As for degree of inflammation in IBD even for asymptomatic individuals, mucosal integrity is gradually altered, even in remission exhibiting high levels of cachexine and intraepithelial lymphocytes (IELs) [91]. In IBS however, they may be less intense or even absent [84]. Skin-antimicrobial peptide 1 (SAP1) is another indicative marker for inflammation, in humans being encoded by the *DEFB4* (*defensin beta 4*) gene. This peptide produced by colonic tissue targeting Gram-negative or -positive overpopulation of viruses, fungi, or bacteria as part of the immune system. In ulcerative colitis, lactoferrin and calprotectin levels were measured with ELISA, while in IBS HBD-2 was determined as well with ELISA, the results indicating a significant increase of HBD-2 in IBS patients compared with controls, the authors considering this finding supportive of the proinflammatory potential [92]. 

Moreover, Caviglia et al. [93] discussed the role of haptoglobin-2 precursor, zonulin, in gut permeability and inflammation considering its implication in tight junction action. In this way, antigens could easily be transported through the intestinal barrier leading to immune activation. Furthermore, Singh et al. [94] found increased serum zonulin levels in IBS patients, comparable to those in celiac disease and a significant correlation between zonulin levels and bowel habit severity in IBS-D. Thus, serum or stool zonulin levels, an alternative non-invasive tool to investigate the integrity of the intestinal barrier, could also be considered a potential IBS molecular marker.

Similar findings are discussed by Fagoonee et al. [95], thus one could be tempted to further suggest that the proinflammatory processes in IBS could be correlated with changes in both intestinal cell inflammation and microbiota changes which could trigger modified immune response. 

Analogous as CRP, ESR is speculated to be a nonspecific indicator for inflammation. Hauser et al. [96] saw an advantage in using erythrocyte sedimentation rate because of its simplicity and low costs. Their pilot study revealed in IBS patients with high expression of ESR, lower disease-specific Health-Related Quality of Life (HRQOL). Cortisol, produced by zona fasciculata in the adrenal cortex, mainly depends upon the integrity of HPA axis. Exposure to chronic stress or trauma at an early age, can be a plausible argument of proceeding possible studies in this context [97,98]. Parathyroid secretory protein 1 is a representative protein which serves as a precursor for peptides with distinct bioactive function [99], presently, chromogranin A being used as marker for neuroendocrine tumors [100]. Recently, a novel biomarker panel for IBS patients has been described with 88.1% sensitivity and 86.5% specificity which could help us in the future to apply to a larger scale [101].

An increased mucosal permeability could explain to some extent visceral hypersensitivity and interactions with enteric nervous system (ENS) that leads to an aggravated symptomatology in IBS [85]. Based on the aspects detailed earlier and results obtained in this context, IBS is not a precursor of colorectal cancer (CRC), data sustained after the analysis of variable cohorts in number (between 30,000 and 90,000 individuals) [86,87,88]. In this way, we find arguments in explaining the main difference between IBS and IBD in which the antioxidant system is impaired and inflammation escalades [98].

Regarding the similarities which can be found between affective disorders and IBS in terms of inflammatory processes, a recent study mentioned that a possible correlation could arise from the influence of stress on colonic permeability, and mucosal and systemic inflammation’s mediation by the autonomic nervous system and the hypothalamo-pituitary-adrenal axis [102]. In this way, while a recent meta-analysis showed a correlative state between IBS-C and IBS-D for anxiety and IBS-D for depression [103], Mudyanadzo et al. [104] suggested that the low-grade inflammation which was previously described in IBS could both impair gut microbiota, but also modify the HPA axis modulatory processes and serotonin reuptake.

## 6. Oxidative Way of Thinking 

Increasing evidence supports a new emerging theory regarding IBS etiology. The fact that oxidative stress is involved in many common physiological pathways and also in highly incident pathologies such as gastrointestinal pathologies, nutritional pathologies, neurological and psychiatric pathologies is not new to research efforts [66]. Since IBS could be the matter of deficient signaling pathways involving both gastrointestinal secretion and neuro-vegetative stimulation, IBS makes no exception from the oxidative hypothesis in the pathological mechanisms.

In a recent review on IBS animal models, Wang et al. [105] noted that the underlying pathogenic mechanisms of IBS remain ambiguous, although increased epithelial permeability, inflammation, visceral hypersensitivity, and altered brain–gut interaction are thought to play an essential role. However, the most used method to obtain IBS symptomatology in animal models is stress exposure despite the action sites of stimuli. This could suggest that stress response pathways could be involved in IBS symptomatology disregarding IBS subtypes. Nevertheless, research efforts confirmed that even in these animal models’ molecular changes occur at systemic and local levels of action. Therefore, it seems that IBS animal models demonstrate that clinical diagnostic and assessment would not be enough in IBS research and treatment.

Regarding the oxidative stress implication in IBS, the previous research efforts showed controversial results. While some animal models and patients’ studies reported clear oxidative imbalance both in systemic and local levels, no concrete evidence points to a direct correlation between oxidative stress and IBS. In this way, Preidis et al. [106] described common oxidative stress patterns in IBS patients such as increased systemic protein and lipid peroxidation, but no impaired glutathione antioxidant pathway, as compared to healthy controls. This could partly explain why IBS was characterized to be only a mild inflammatory imbalance, since a good working antioxidant system means a good working anti-inflammatory system.

The correlation between antioxidant system and IBS was further established in animal model studies. In this way, the link between inflammation and increased harmful reactive oxygen species (ROS) was made in an *Aloe vera* and *Matricaria recutita* mixture antioxidant potential study that aimed to describe its beneficial effects on gastrointestinal imbalance induced by contention-stress [107]. Moreover, they observed that the administration of plant mixture and spasmolytic agents changed the oxidative stress imbalance in colonic cells together with antioxidant capacity improvement, but with no evidence on the correlation type between harmful events and antioxidant defense. Similar studies also reported links between smooth muscles activity and total antioxidant capacity in a wrap-restrained IBS model [72] and oxidative stress and contention-stress IBS model [108], choline precursor and intestinal oxidative signaling in acetic acid-induced IBS rat model [109], circadian rhythm hormone and systemic oxidative stress in noise-stress gastrointestinal distress rat model [73]. In this way, our recent results showed that in a contention-stress IBS model, brain oxidative stress processes are significantly correlated to behavioral parameters suggesting that strong connection between the digestive system, enteric nervous system, and the central nervous system lead to the known IBS symptoms [108].

However, none of these studies presented reasonable evidence on the molecular pathways connecting oxidative stress to gastrointestinal imbalance observed and diagnosed as IBS. This suggested that the current research efforts are neither supportive nor disproving regarding the hypothesis of oxidative stress origin of IBS which bring us back to human patient studies. According to Choghakhori et al. [77] observations, it appears that although there are differences in IBS patient’s serum oxidative markers, as compared to healthy controls, these variations are not directly correlated to the digestive symptoms or the quality of life. By contrast, the authors noted significant correlations between the inflammatory parameters and the latter. 

Therefore, considering that inflammation and oxidative stress form a circuit acting in tandem, as the authors argue, we could discuss the implication of oxidative stress in IBS not as a pathological development factor but as a ripple effect of the true molecular origin of IBS. Similarly, Oran et al. [65] suggested that IBS and oxidative stress are associated via the micro-inflammatory processes. They pointed to the definition of oxidative stress being the effect of antioxidant defenses overwhelmed by ROS, however they attributed to ROS the pro-inflammatory mediation role which is overplayed due to their excess production and deficient hydrolysis. 

Since conjugated diene levels assessed by Oran et al. [65] in IBS patients’ blood samples is an indicator of increased inflammation and also lipid peroxidation, they explained the link between the oxidative stress harmful events and inflammatory processes which further could occur, with evidence that ROS overproduction is due to inflammatory signaling originating in colonic cells of the IBS patients.

Mete et al. [63], however, thoroughly discussed the oxidative and nitrosative changes in IBS patients in relation to some of the gastrointestinal symptomatology. In this way, starting with the report regarding the significant decreases in SOD, GPX, and CAT antioxidant enzyme activities, together with the mild increase in activity of XO and AD, they presented rational argumentation of NO implication in IBS-C subtype, based on the smooth muscle contraction inhibitory property of NO. Moreover, regarding the duality of NO implication in inflammation by relation to normal and abnormal physiological conditions [64], it is fair to consider these oxidative and nitrosative changes in inflammatory circumstances when discussing IBS pathogenesis.

## 7. Is It Written in Our Genes?

The Human Genome Project (HGP) enabled the entry into the land of the “omics” by providing a new perspective on the role of genes in human health and disease. While there are still many pieces of the puzzle that must be put in “post-genome” era, the idea of a gene that causes IBS is appealing. Although it is known that aberrations such as cancer always have a deregulation of the cell cycle mechanism, of the checkpoints p53/pRb, or due to chromosomal discrepancies, the classical Mendelian model also enters the scene, circulating speculations according to which IBS has a hereditary component. This argument is sustained by a Swedish nationwide study that included more than 50,000 reports in first-, second-, and third-degree relatives who clearly highlight the involvement of a genetic component [110].

While traditional genetic abnormalities caused by a mutation of one or few mutations in a single gene who were transmitted either as autosomal dominant or in a recessive manner, IBS is regarded as a mixture of psychosocial and exogenous factors. Saito et al. [111] discussed the multifactorial and multi-interactional character of IBS, with risk factors such as the traumas experienced in the army, verbal, physical or sexual abuse, socioeconomic status, unemployment, parent’s lifestyle, painful stimuli exposure during childhood, or who have suffered from a natural disaster.

Recent reports showed an interesting correlation between the gene/environment interactions with IBS occurrence. Thus, gender, neurobiology, immunity, and personality genes may fully interact with environment factors such as diet, stress, trauma, and infections leading to a molecular context in which IBS types may occur as a phenotypization result. Moreover, the concept of familial IBS was newly introduced being described as a functional gastrointestinal disorder resembling IBS symptomatology and being passed through the generations [14]. Thus, some major gastrointestinal disease susceptibility genes were tested against IBS occurrence including several serotonin receptors genes, several inflammatory pathways genes, and several known loci involved in affective disorders. Unfortunately, only few variants of the tested genes were found to be correlated with IBS. It seems that only HTR2A, HTR3E, IL10, and IL6 may possess intriguing potential as candidate IBS loci. Regarding these genes, IL10 and IL8 could of course play important roles in oxidative stress regulation. In this way, the polymorphisms of the 5-HTR3 receptor, subunit A, subunit C-42 C>T and 5-HTR2 receptor subunit A, -1438(G/A) and 102 T/C, extensively reported as implicated in IBS predisposition and often associated with bloating, intense pain, and increased anxiety in IBS patients, will be correlated with presence of IL-8 ATCC haplotypes (at positions −251, +396, +781, and +1633) and IL 10 ACC haplotypes (at positions −1082, −819, and −592) in IBS patients. Moreover, tryptophan hydroxylase genes expression in TPH isoforms (TPH1 and TPH2) could be analyzed as well as tumor necrosis factor alpha polymorphism at positions −308 and −238 which have all shown to be implicated in IBS predisposition. Moreover, some of these variants were also described to be implicated in affective disorders [112,113].

One major step was represented by the discovery of the NOD2/CARD15 (nucleotide-binding oligomerization domain protein 2/caspase recruitment domain-containing protein 15) gene for Crohn’s disease [92,100]. A combination of family-based linkage, candidate genes, and mapping studies led to the identification of several others like autophagy related 16 like 1 (ATG16L1), interleukin-23 (IL-23), natural killer cell stimulatory factor 2, signal transducer and activator of transcription 3 (STAT3), and home-box protein Nkx-2.3 (NKX2-3) [110]. To date a considerable number of genes for IBS have undergone a thorough examination, and according to the best evidence of association with IBS are summarized in Table 2.

With the presumption that bile acid malabsorption of moderate severity might play a role in IBS-D, Wong et al. [114] tested 435 IBS subjects and 270 healthy controls and found out that a functional KLB gene variant is involved in the modulation of protein stability associated with colonic transit in IBS-D, association mediated by two genetic variants in FGFR4 (FGF19-FGFR4-KLB pathway). One of the strongest risk factors for IBS, acute gastroenteritis, was also targeted, the authors comparing residents who developed gastroenteritis with those who do not and revealed that TLR9, IL-6, and CHD1 persisted in all situations as a risk factor for PI-IBS out of all 228 cases [115]. 

Due to the complex etiology of IBS, two independent groups were genotyped in order to determine whether there are genetic variabilities in immune, neuronal, and barrier integrity, two single nucleotide polymorphisms being revealed: rs17837965-CDC42 for IBS-C and rs2349775-NXPH1 for IBS-D [120]. A genome-wide association study was conducted in the Swedish general population from which was deducted that two genes (KDELR2—KDEL endoplasmic reticulum protein retention receptor 2) and (GRIP2IP—glutamate receptor, ionotropic, delta 2 (Grid2) interacting protein) showed consistent effects for IBS [118]. As for IBS-C, from 584 patients, about 2% of them carry mutations in the SCN5A gene, as for TNFSF15 member, TL1A is a participant in the modulation of inflammatory responses [116,117]. The first evidence for a link between serotoninergic receptors (5-TH_3_—5-HT_3A_ and 5-HT_3E_) that for female IBS-D remains a cis-regulatory mechanism which resides on two functional variants—HTR3E c.76G.A for microRNA and HTR3A c.-42C.T—was also highlighted [119].

Despite the concern of other genetic disorders that mimic IBS, Saito et al. [111] revealed that in their study of over 477 cases IBS aggregates strongly in families. When Villani et al. [121] screened loci linked to ulcerative colitis and Crohn’s disease, those for IBD were not observed in IBS. However, they provide a roadmap by comparing risk factors for PI-IBS and sporadic IBS in which toll-like receptor 9, cadherin-1, and interleukin 6 appeared to be involved in PI-IBS, while for sporadic IBS, genetic risk factors were represented by a deregulation of serotoninergic pathway of serotonin receptor 2A and solute carrier family 6 (neurotransmitter transporter, serotonin), member 4 and the secretion of interleukin 10. Like the previous case, lactase non persistence did not differ notably (15.1%–14.8%). This suggests that this autosomal recessive feature does not explain IBS and familial aggregation [122].

Whereas almost 100% of total serotonin is stored in enterochromaffin cells, selective serotonin reuptake inhibitors (SSRIs) are the most effective anti-depressant, serotonin agonists and antagonists being able to accelerate or slow GI transit [123]. Even though 5-HTT LPR variable number tandem repeat (VNTR) polymorphism is the best functional variant (promoter of region SLC6A4) in IBS, from all the potential candidate genes, a clear relationship between VNTR and IBS does not exist [124]. 

Fukudo et al. [125] studied the polymorphism of 5-HT3B and found out that distinctive regions of the brain are activated between A/A, A/C, and C/C. Mood disorders like depression and anxiety were associated with IBS. More precisely, COMT Val158Met variant is related to IBS and OPRM1 118A>G variant could predict placebo effects [126,127]. Even BDNF Val166Met SNP was associated with IBS, especially for those with psychiatric disorders [128]. 

Brain-derived neurotrophic factor (BDNF), a key modulator of brain’s development whose activity is influenced by genetic or environmental factors in individuals with schizophrenia, BDNF Val66Met SNP is another variant linked with IBS [128], while OPRM1 to pain and social sensitivity and opioid appurtenance [129]. 

In addition, a team of researchers first studied extensively GI transit and postulated that serotoninergic and adrenergic mechanisms modulate GI motility which is why they performed an exploratory study for four functional variants ADRA2A −1291C>G, ADRC2A Del 332–325, GNB3 825C>T, and 5-HTT LPR. However, no direct correlation was found, but GNβ3 CC appeared to be an indicator for IBS-D and ADRA2A CC genotype was predictive for IBS-M [130,131].

## 8. The Future Is Now

According to the improved ROME IV, IBS diagnosis criteria newly include the neurologic and affective component. Thus, it seems that the correlation between the brain and gut gained new features according to the aspects described by the relevant recent studies. The fact that the molecular biomarkers available to date can only offer a non-IBS diagnostic (differential diagnosis based on negative result to clinical/molecular biomarker) rather than diagnose or predict IBS together with the fact that several genetical resemblances with affective spectrum disorders were already described lead to the assumption that IBS could in fact be more likely a stress disorder rather than a gastrointestinal one. In this way, efforts are made to identify specific non-invasive biomarkers from different body fluids considering a strong link with neurological and psychiatric components. Moreover, in the light of the novel description of the brain–gut axis, HPS axis, and microbiome–brain interaction in IBS, further studies could consider the implication of oxidative stress and inflammation in IBS from a central or peripheric mediation point of view.

## 9. Conclusions

No current valid molecular biomarker was identified for IBS. However, several hypotheses suggest a complex interaction between its molecular features and rich similarities to affective spectrum disorders. The present knowledge in IBS pathophysiology gives oxidative stress, inflammation, genetic landscape, and gut microbiota determinant roles, but still it is the complex interaction between these components that outlines the multifactorial character of IBS, its variable clinical panel, and insufficiently understood pathological mechanisms. Moreover, the genetic features study in IBS patients showed that several genetic similarities point to a possible correlation of IBS with affective spectrum disorders. Thus, we open here the discussion on the assumption that IBS could in fact be more likely a stress-related disorder rather than a gastrointestinal one.

## Figures and Tables

**Table 1:** Changes occurred at level of gut flora diversity in different subtypes of irritable bowel syndrome (IBS).

Microbiota Diversity	Microbiota Alterations	IBS Subtypes	References
*Clostridiales*/*Bacteroides*, *Prevotella*	↑/↓	IBS-C	[55]
*Ruminococcus bromii*-like phylotype	↑	IBS-C	[29]
*Veilonella* and *Lactobacillus* spp.	↑	IBS-C	[30]
*Bifidobacterium catenulatum*	↓	IBS-C	[31]
*Methanobrevibacter smithii*	↑	IBS-C	[32]
unknown *Ruminococcaceae* and *Christensenellaceae*, *Akkermansia*, *Methanobrevibacter*	↑	IBS-C	[56]
*Lachnospira*, *Parasutterella*, *Lactobacillus*, *Turicibacter*, *Enterococcus*, *Weissella*, *Oxalobacter*, *Oceanobacillus*, *Lachnospiraceae*_UCG-010/NK4A136_group and *Ruminococcaceae*_UCG-003/*Faecalitalea* and *Prevotella*	↓/↑	IBS-D	[43]
*Clostridiales* and *Bacteroides*/*Prevotella*	↑/↓	IBS-D	[55]
*Clostridium thermosuccinogenes* and *Ruminococcus torques*/*Collinsella aerofaciens*, *Bacteroides intestinalis*	85% and 94% phylotype ↑/↓	IBS-D	[29]
*Lactobacillus* spp.	↓	IBS-D	[30]
*Bifidobacterium catenulatum*	↓	IBS-D	[31]
*Enterobacteriaceae*	↑	IBS-D	[33]
*Lactobacillus* spp.	↑	IBS-D	[34]
*Ruminococcaceae*, unknown *Clostridiales*, *Erysipelotrichaceae* and *Methanobacteriaceae*	↓	ISB-D	[56]

**Table 2:** IBS-risk genes prioritized based on existing studies.

Gene	Gene Name	Region	Chromosome	Phenotype	References
*KLB*	*Klotho Beta-Like Protein*	Coding polymorphism	4p14	IBS-D	[114]
*TLR-9*	*Toll-like receptor 9*	Intron and upstream	3p21.3	PI-IBS	[115]
*SCN5A*	*Sodium channel protein, cardiac muscle alpha-subunit*	Rare coding mutations	3p21	IBS, IBS-C	[116]
*TNFSF15*	*Tumor Necrosis Factor Ligand Superfamily, Member 15*	Intron	9p32	IBS, IBS-C	[117]
*KDELR2*	*(Lys-Asp-Glu-Leu) Endoplasmic Reticulum Protein Retention Receptor 2*	Intron	7p22.1	IBS	[118]
*HTR3E*	*5-Hydroxytryptamine (Serotonin) Receptor 3, Family Member E*	3’-untranslated region (3’-UTR)	3q27.1	IBS-D	[119]
*CDC42*	*Cell division control protein 42 homolog*	Intron	1p36.1	IBS-C	[120]

KLB = *Klotho Beta-Like Protein*, *TLR-9* = *Toll-like receptor 9*, *SCN5A* = *Sodium channel protein*, *cardiac muscle alpha-subunit*, *TNFSF15* = *Tumor Necrosis Factor Ligand Superfamily*, *Member 15, KDELR2* = *(Lys-Asp-Glu-Leu) Endoplasmic Reticulum Protein Retention Receptor 2*, *HTR3E* = *5-Hydroxytryptamine (Serotonin) Receptor 3*, *Family Member E*, *CDC42* = *Cell division control protein 42 homolog*, IBS-D = diarrhea-predominant irritable bowel syndrome, PI-IBS = post infectious irritable bowel syndrome, IBS-C = constipation-predominant irritable bowel syndrome.
